# Classification, Toxicity and Bioactivity of Natural Diterpenoid Alkaloids

**DOI:** 10.3390/molecules26134103

**Published:** 2021-07-05

**Authors:** Amin Mahmood Thawabteh, Alà Thawabteh, Filomena Lelario, Sabino Aurelio Bufo, Laura Scrano

**Affiliations:** 1 Samih Darwazah Institute for Pharmaceutical Industries, Faculty of Pharmacy Nursing and Health Professions, Birzeit University, Bir Zeit 71939, Palestine; athawabtah@birzeit.edu; 2 Medical Imaging Department, Faculty of Health Profession, Al-Quds University, Jerusalem 20002, Palestine; athawabteh@staff.alquds.edu; 3 Department of Sciences, University of Basilicata, 85100 Potenza, Italy; filomena.lelario@unibas.it; 4 Department of Geography, Environmental Management & Energy Studies, University of Johannesburg, Johannesburg 2092, South Africa; 5 Department of European Cultures (DICEM), University of Basilicata, 75100 Matera, Italy; laura.scrano@unibas.it

**Keywords:** diterpenoid alkaloids, *Aconitum*, *Delphinium*, *Consolida*, structural substituents, marine sponges, bioactivity, toxicity

## Abstract

Diterpenoid alkaloids are natural compounds having complex structural features with many stereo-centres originating from the amination of natural tetracyclic diterpenes and produced primarily from plants in the *Aconitum*, *Delphinium*, *Consolida* genera. Corals, *Xenia*, Okinawan/*Clavularia*, Alcyonacea (soft corals) and marine sponges are rich sources of diterpenoids, despite the difficulty to access them and the lack of availability. Researchers have long been concerned with the potential beneficial or harmful effects of diterpenoid alkaloids due to their structural complexity, which accounts for their use as pharmaceuticals as well as their lousy reputation as toxic substances. Compounds belonging to this unique and fascinating family of natural products exhibit a broad spectrum of biological activities. Some of these compounds are on the list of clinical drugs, while others act as incredibly potent neurotoxins. Despite numerous attempts to prepare synthetic products, this review only introduces the natural diterpenoid alkaloids, describing ‘compounds’ structures and classifications and their toxicity and bioactivity. The purpose of the review is to highlight some existing relationships between the presence of substituents in the structure of such molecules and their recognised bioactivity.

## 1. Introduction

Diterpenoid alkaloids (DAs) are substances produced by various natural plants with significant thematic difficulties, bioactivity, and somewhat disreputable toxicity. To date, 1500 and more DAs have been isolated and characterised. Many remarkable DAs demonstrate different pharmacological properties such as neurotropic, antimicrobial, antitumour, hypotensive, analgesic, anti-inflammatory, muscle relaxant, antiarrhythmic, and local anaesthetic [1,2,3,4,5,6,7,8].

DAs extracted from some plants belonging to the Ranunculaceae family, especially genera *Delphinium*, *Aconitum* and *Consolida*, are often distinctive and recognised as cytotoxic against cancer [9]. *Aconitum* spp. (monkshood) is one of the most extracted and isolated sources of DAs, where more than half of natural DAs were isolated from [10,11,12,13,14,15].

DAs have long been used all over the world. People extract *Aconitum* due to either medicinal and beneficial properties or toxic and harmful ones. In the ancient past, *Aconitum* seasoned the top of arrows used for hunting animals and in wars. Of note, a Chinese tribe discovered that extracted crystallised *Aconitum* turned into a sand-like substance when left for some time [16,17,18,19].

The first DA was isolated by Geiger in the early nineteenth century when he isolated aconitine from *Aconitum napellus*. Then followed growing success in isolating many other DAs with simultaneous development of extraction methods, purification techniques and molecular identification, favouring their widespread use in medicine and scientific research [1,3,7,9].

In the last ten years, we can acknowledge significant progress in studying DAs’ phytochemistry and identifying new natural DAs. Numerous researches performed are the nourishment of many scientific articles and reviews on phytochemistry, chemical reactions, compositional and botanical studies, and ‘DAs’ biological activities, classifying these substances as structures containing either 18 or 19 and 20 cycled carbon atoms (C_18_, C_19_, C_20_), i.e., according to the number of contiguous carbon atoms that constitute their central arrangement [20,21].

The purpose of this review is to highlight some existing relationships between the presence of substituents in the structure of such molecules and their recognised bioactivity. Structure–activity relationship (SAR) analysis can guide researchers to modify existing natural molecules or synthesise new compounds to propose novel-effective drugs. Furthermore, for comparison with plant extracted DAs, a section of this review highlights the structure of marine-diterpenoids, most of which are not alkaloids but, mainly used in alternative medicine, having received significant attention by researchers in the last decade.

## 2. Classification of Diterpenoid Alkaloids

Diterpenoid alkaloids are heterocyclic systems containing β-aminoethanol, methylamine, or ethylamine nitrogen atoms derived from the amination of tetra- or pentacyclic diterpenoids and classified into C_18_-, C_19_-, and C_20_-diterpenoid alkaloids, according to their carbon skeleton configuration [7].

### 2.1. C_18_-Diterpenoid Alkaloids

These compounds, for a long time structurally classified as belonging to the broad group of C_19_-diterpenoid alkaloids, are currently considered independent. Furthermore, they are split into two distinct types: the ranaconitine-type and the lappaconitine-type. The main difference between them is the presence of additional oxygenation on the C7 position in the ranaconitine-type.

#### 2.1.1. Ranaconitine Group

**Figure 1:**
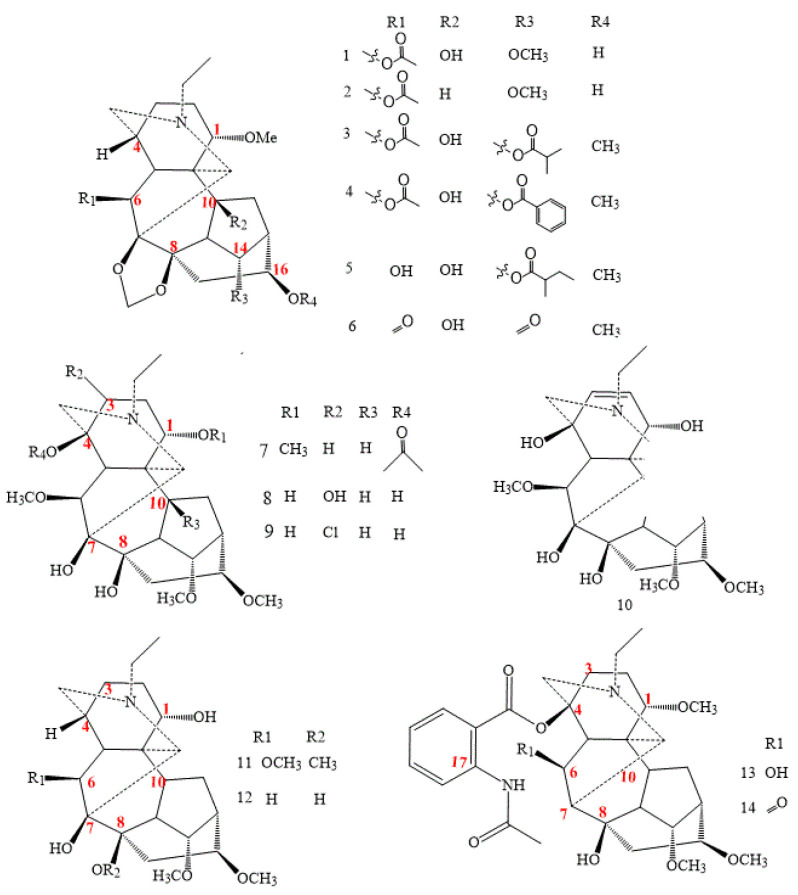
C_18_-ranaconitine Das.

The majority of this class of compounds comes from *Aconitum* plants and others from *Delphinium*. In this class, alkaloid compounds having an oxygen group functionality in C7 comprise more than ten new compounds (structures **1–14** in Figure 1) [5,6,7,8].

Compounds **1–6** contain a 7,8-methylenedioxy group, while compounds **1**, **3–6** feature a 10-OH. In particular, alkaloids 1–2 have a 16-OH group instead of the O-methyl unit, usual in C_18_-DAs [22,23,24,25,26].

The bi-hydroxyl groups distinguish the compounds (**7–12**) in C8 and C7 [27]. Compound **9**—puberumine—is the first example of a naturally occurring DA containing the chlorine substitute on C3 [27].

On the other hand, compound **10** in Figure 1 has a double bond in C2-C3, which distinguishes it from the rest of the group, and vaginatunine (**11**, in Figure 1) shows the presence of a methoxy substitute in C8 [28].

Alkaloids **13** and **14** in Figure 1 have an N-acetylanthranoyloxy substituent in C17; furthermore, they have fewer ester groups, suggesting that they are less toxic [29].

#### 2.1.2. Lappaconitine Group

The methine unit in C7 characterises the lappaconitine compounds; examples are natural to various *Aconitum* and *Delphinium* species [5,8].

**Figure 2:**
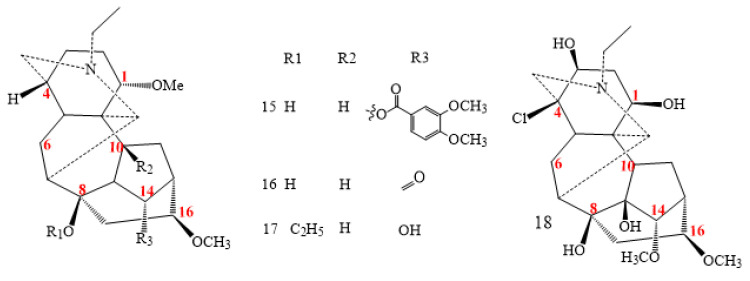
C_18_-lappaconitine DAs.

Minor toxicity characterises plant roots containing weisaconitine compounds (structures **15–18** in Figure 2) [30]. Structurally, the lactam carbonyl and acetoxy groups are specific to weisaconitine compounds **16** and **17**, respectively. Furthermore, a chloro-substitution in C4 and two hydroxyl groups in C1 and C3 are natural to sinomontanine N (**18**) [31].

#### 2.1.3. Rearranged Group

**Figure 3:**
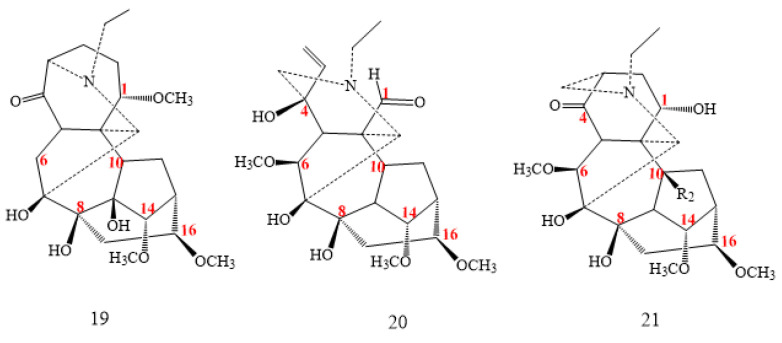
C_18_-rearranged DAs.

Sinomontadine (**19** in Figure 3), isolated from *Aconitum sinomontanum*, shows a surpassing skeleton unlike other DAs compounds; it exhibits a seven-membered ring [31] instead of six, by the incorporation of a carbon atom into the six-ring system with the expansion to a seven carbons ring. The result is a set of new compounds such as puberudine (**20**, Figure 3) and puberunine (**21**, Figure 3), isolated from *Aconitum barbatum* var. *puberulum* and recognised as an exceptional class of DAs [27].

Puberudine (compound **20** in Figure 3) has a distinctive characteristic in the A ring, which is an open ring (1,2-Seco), and also a specific double bond between C2 and C3, in addition to the carbonyl group on C1 instead of the methoxyl or hydroxyl group [27,31].

### 2.2. C_19_-Diterpenoid Alkaloids

C_19_- is the largest category of the DAs, belonging to pentacyclic compounds. Most of the C_19_-DAs are isolated from *Aconitum*, *Delphinium*, and the roots of *Aconitum carmichaelii* [9].

**Figure 4:**
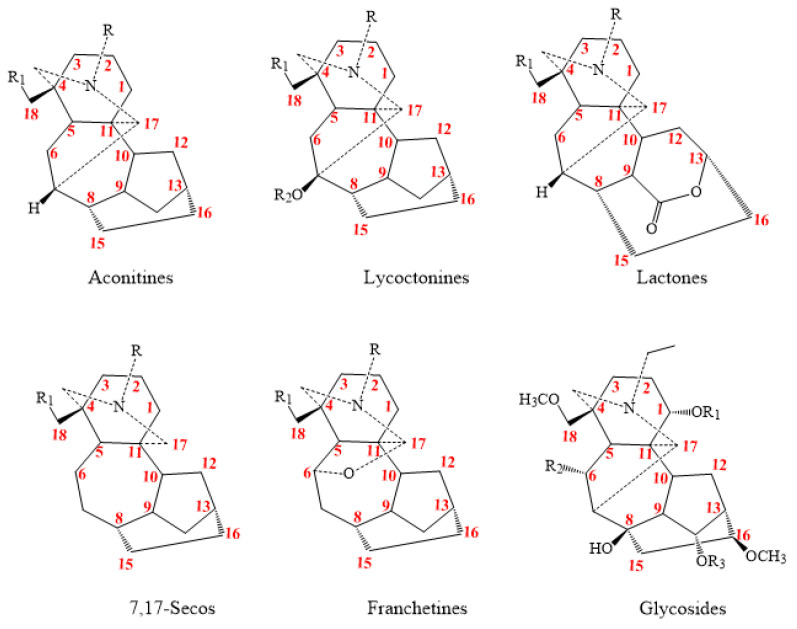
C_19_-DAs class.

C_19_-DAs are compounds classified into seven types (lactone, aconitine, lycoctonine, 7,17-Seco, franchetine, rearranged class, and glycosides) according to the oxygen-containing groups on C7 and the difference of skeleton as shown in Figure 4 [20,21].

The plurality of C_19_-DAs are lycoctonine and aconitine types, which are isolated from *Delphinium*. The presence of the oxygen-substituent group in the lycoctonine-type on C7 constitutes the difference between them.

#### 2.2.1. C_19_-Aconitine Class

**Figure 5:**
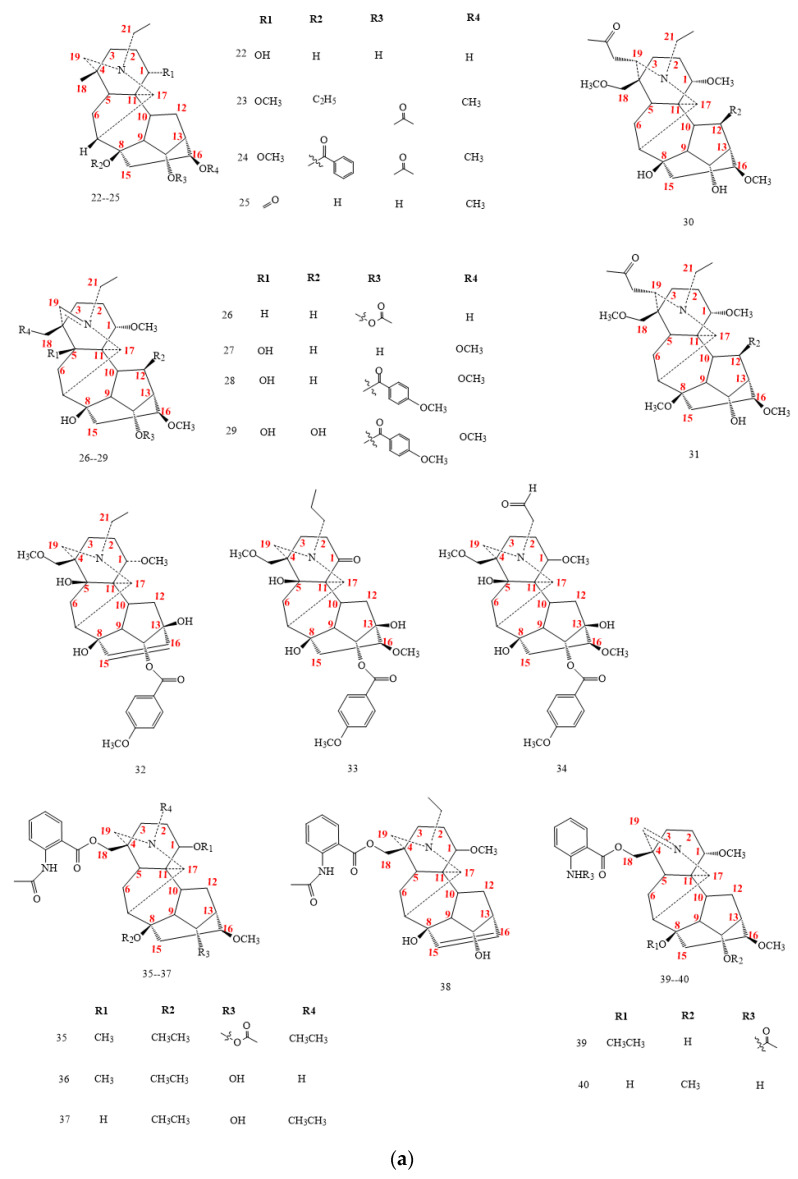

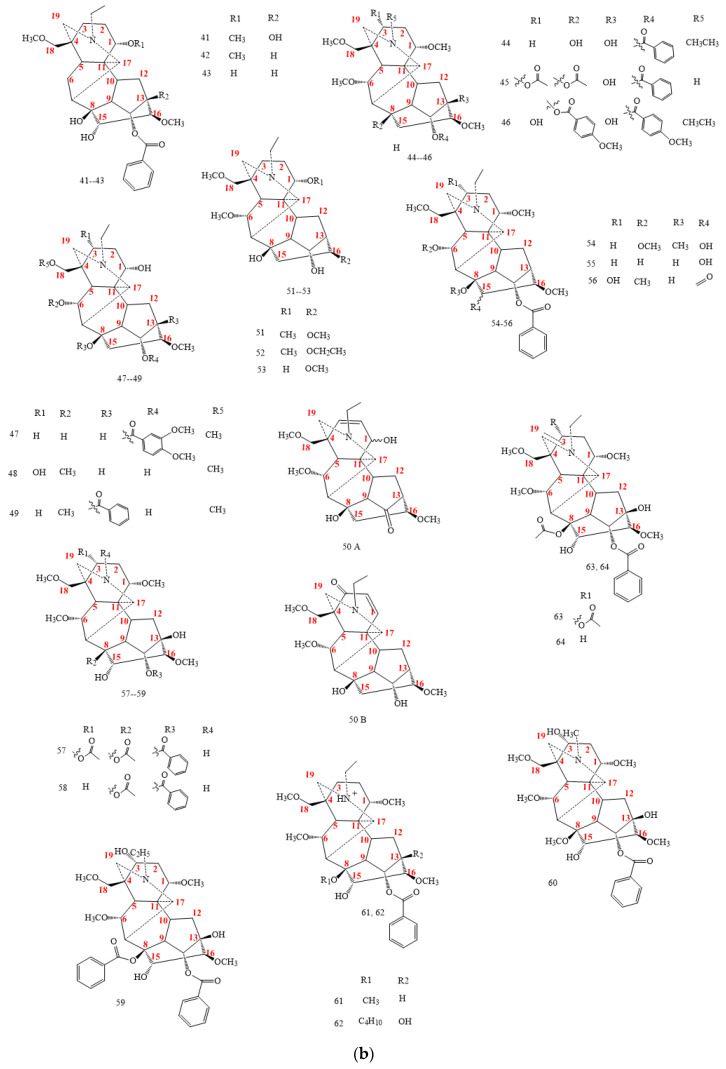
(**a**) C_19_-aconitine Das, structures **22–40**. (**b**) C_19_-aconitine Das, structures **41–62**.

Aconitines (structures **22–62** in Figure 5a,b) do not show an oxygenated C7. Due to the ester-group presence on C8 and sometimes on C14, they exhibit acute toxicity [1,3,8]. Several aconitines lack oxygenated groups on C15 and C6 (**22–40**) and rarely arrange a hydroxyl group on C1 as compound **22** [2,3,32,33,34,35,36,37,38,39,40,41,42,43,44,45,46,47,48,49,50].

Some aconitine compounds (**26–29**, in Figure 5a) display a double bond between C19 and N, which isolate from *Aconitum hemsleyanum* var. *circinatum* and *Aconitum straminifiorum*. Others bear an additional CH_2_COCH_3_ group on C19, with the same skeleton as acetonyl-talatisamine (**30**, Figure 5a) and hemaconitine D (**31**, Figure 5a) [41,42,51].

Three new C_19_-DA compounds, isolated from the genus *Aconitum* (**32–34**), distinguish an anisoyl group in C14 and a double bond between C15 and C16, as for compound **32**. DAs **35–40** have an anthranoyl substituent in C18 and a double bond between C15 and C16, as is visible for compounds **38–40**, further to the specific double bond N=C19 [37,38]. Compounds **41–43** in Figure 5b are water extract of the *A. carmichaelii* lateral roots. They lack the oxygenated unit in C6 while showing an oxygenated group in C15. On the contrary, other C19-DAs (**44–53**) have an oxygen-containing substituent in C6 and lack oxygen in C15. And some (**54–64**) have both oxygen-containing groups in C6 and C15 [3,32].

Furthermore, DAs **61–62**, isolated from the roots of *A. carmichaelii*, are characterised by the presence of quaternary amine (cation) having a positive charge (+HN-3R), which tolerate a function similar to that of a nitrone (+NO=C) [52].

#### 2.2.2. C_19_-Lycoctonine Class

**Figure 6:**
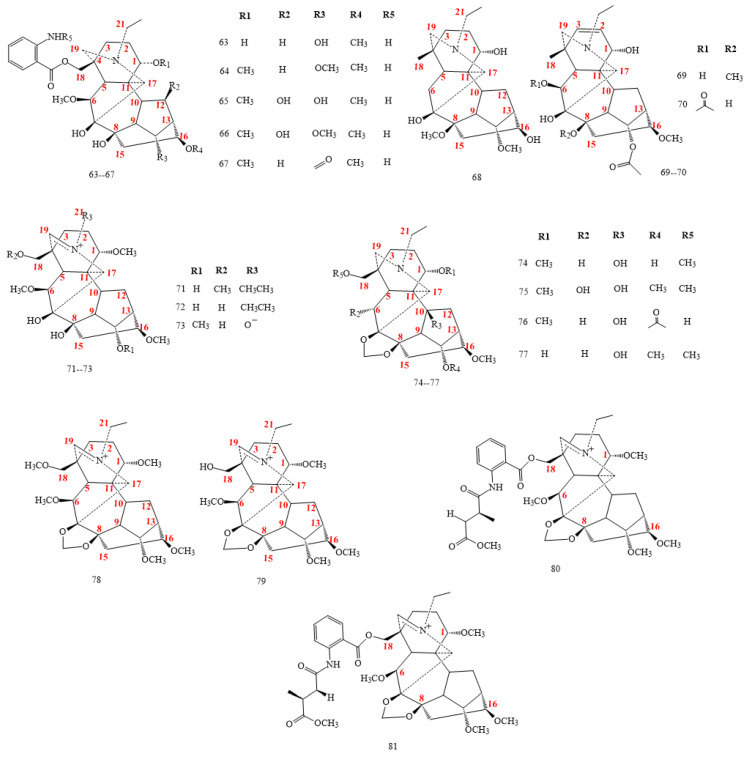
C_19_-lycoctonine DAs.

The ester ratio at C8 or C14 in lycoctonines (Figure 6) is less than the ester ratio in aconitine, whereas the ratio at C18 is higher in the lycoctonine class. Lycoctonines are subdivide into two subtypes based on the methylenedioxy group attached at C7 and C8. Some lycoctonines, isolated from *Aconitum*, differentiate with diol at C7-C8 as compounds **63–73** in Figure 6. Other lycoctonines characterise the presence of 7,8 methylenedioxy group (**74–81**), as shown in Figure 6 [22,53,54,55,56,57,58,59,60,61,62,63].

DAs **63–67** in Figure 6 show an O-acetamidobenzoate moiety [54,55,56,57]. DAs 65 and 66 exhibit hydroxyl substitution at C12 [54]. Tianshanitine B (*68*) has hydroxyl instead of methoxy in C16. Anthriscifoldine A (**69**) and majusine C (**70**) exhibit a double bond between C2 and C3 [22,56,58]. A nitrone functionality is highlightable in DAs 71–73 (Figure 6) due to the N=C19 moiety.

An unfamiliar hydroxyl group is detectable on C10 in compounds **74–77**. Moreover, DAs **78–81** show a quaternary amine with the N=C19 double bond [63,64].

The known, naturally occurring alkaloids of the amine subtypes in the aconitine and lycoctonine types possess the following distinctive features:

(i) In most cases, they have oxygenated functionalities at C1, C6, C8, C14, C16, and C18. Interestingly, the positions of these oxygenated groups are specific for the resulting structural tendency from simple to complex: C13 or C10 to C3/C13 or C3/C10 to C3/C13/C15 or C3/C10/C13/C15 [32,50].
(ii) Many alkaloids contain only the common oxygenated groups, e.g., methoxyl and hydroxyl group(s). In most cases, the methoxyl groups locate at C1, C16, and C18. The hydroxyl groups mainly located at C8 and C14. The presence of hydroxyl groups at C3, C10, C13, and C15 may lead to their structural diversity [53,54,63].
(iii) Some alkaloids contain only the common ester groups, e.g., acetoxy group and benzoyloxy. There are a few examples with other ester groups. Among them, the acetoxy group presents a chemotaxonomic characteristic. The ester groups locate at C8, C14, or C8/C14 [3,32].
(iv) They contain an N-ethyl structural unit. Very few alkaloids possess an N-methyl group [33].
(v) The oxygenated substituents at the C1, C6, and C15 positions of the alkaloids possess an a-orientation in most cases [42].

#### 2.2.3. C_19_-Lactone Class

**Figure 7:**
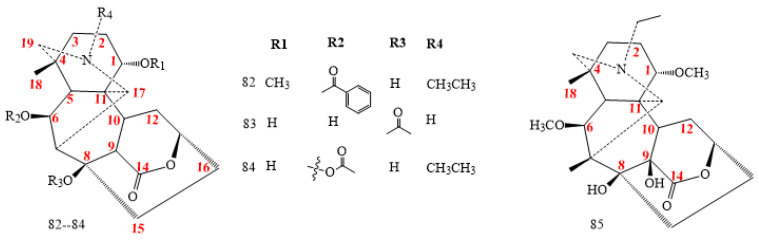
C_19_-lactone DAs.

A six-membered lactone characterises this class (structures **82–85** in Figure 7) obtained by the oxidation of the ketone existing at C14 of aconitine (Figure 7) [65,66,67].

The lactone-type C_19_-diterpenoid alkaloids contain simpler oxygenated functionalities as compared to the aconitine- and lycoctonine-type C_19_-diterpenoid alkaloids. All lactone-type C_19_-diterpenoid alkaloids lack oxygenated functionalities at the C3, C7, C13, C15, and C16 positions and possess oxygenated groups at the C1 and C8 positions. They also have an oxygenated functionality at the C6 position in most cases. Only a very few alkaloids have no oxygenated groups at both C6 and C16 positions [65,66,67].

#### 2.2.4. C_19_-7,17-Seco Class

**Figure 8:**
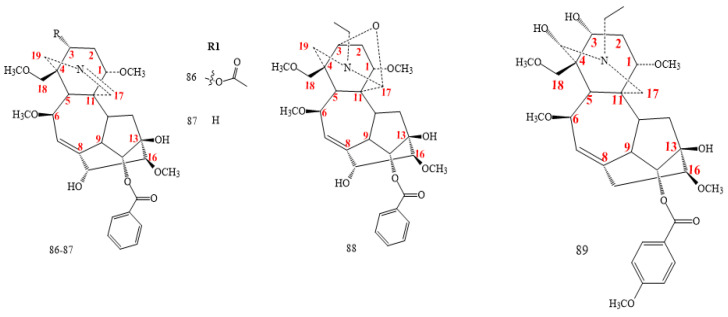
C_19_ 7,17-Seco DAs.

7,17-Seco compounds derive from aconitine DAs with outstanding C7-C8 double bond. DAs **86–89** (Figure 8) show oxygen in C15, except for compound **89**. Most of the Seco DAs class come from *Aconitum brachypodum* [42,68,69,70]. Brachyaconitine C (**86**) exhibits a C17=N unit in 7,17, while secoaconitine (**88**) shows an epoxy ring between C17 and C3 [69].

#### 2.2.5. C_19_-Franchetine Class

**Figure 9:**
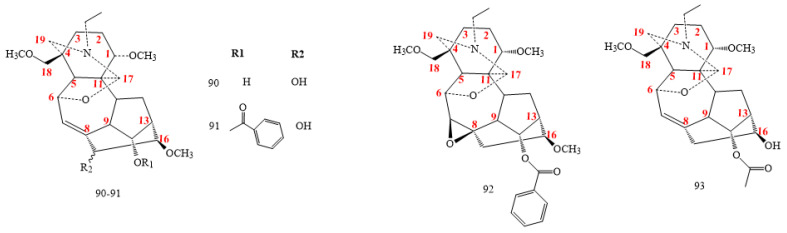
C_19_-franchetine DAs.

DAs in this class (**90–93** in Figure 9) feature an additional oxygenated bond between C6 and C17 [37,38,41,47,71,72]. All compounds exhibit a double bond between C7 and C8, except **92**, 7,8-epoxy-franchetine from *A. straminifiorum* [37,38,42,71,72]. Guiwuline (structure **90** in Figure 9) is an example of a compound having an OH group in C15 [37].

#### 2.2.6. C_19_-DA Glycosides

**Figure 10:**
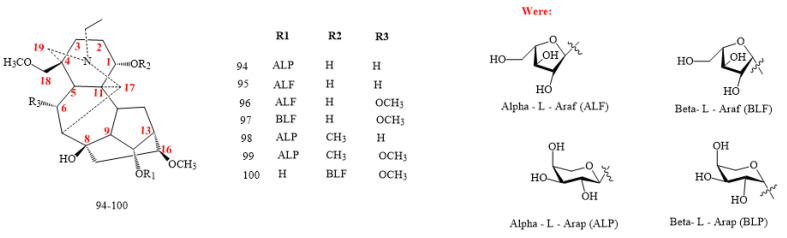
C_19_-glycosides DAs.

Aconicarmichosides A–H, K–L, and I–J (structures **94–100** in Figure 10) are the only glycosidic DAs found in nature [20,21]. Structurally, they belong to the aconitine class, with the addition of the sugar moieties, and include L-arap and L-araf in C1 or C14 [5,6,7,8]. These compounds are currently components of the aqueous extract from *A. carmichaelii* lateral roots [5,6,7,8].

#### 2.2.7. C_19_-DA Rearranged Class

**Figure 11:**
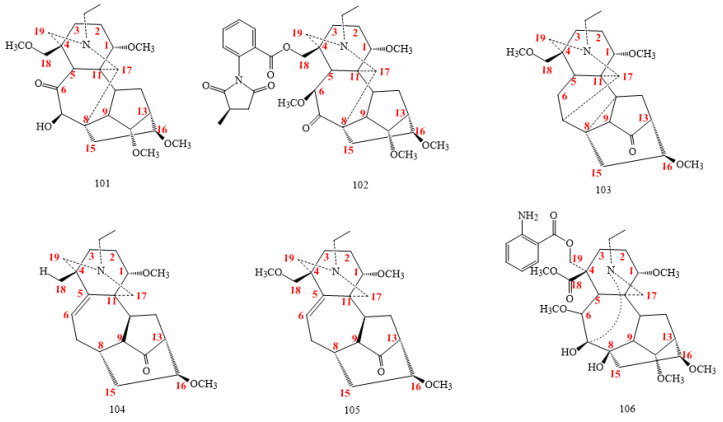
C_19_-rearranged DAs.

Puberuline C and yunnanenseine A (structures **101** and **102** in Figure 11, respectively), isolated in the order from *A. barbatum* var. *puberulum* and *Delphinium yunnanense*, belong to the rearranged class with the C8-C17 bond, rather than a C7-C17 bond [73,74,75].

Aconitramine A (**103** in Figure 11), isolated from the *Aconitum transsectum*, shows a three-membered ring formed via C8, C9, and C10 [32].

Hemsleyaconitines F (**104** in Figure 11) and G (**105** in Figure 11), typically extracted from *A. hemsleyanum*, exhibit skeletons with five-membered D-ring linking C9, C13, C14, C15, and C16, which looks different from the six-membered D-ring of their analogues [74].

Grandiflodine B (compound **106** in Figure 11) from *Delphinium grandiflorum* is distinctive of a remarkable skeleton with the cleavage of N–C19 and C7–C17 bonds [76].

### 2.3. C_20_-Diterpenoid Alkaloids

**Figure 12:**
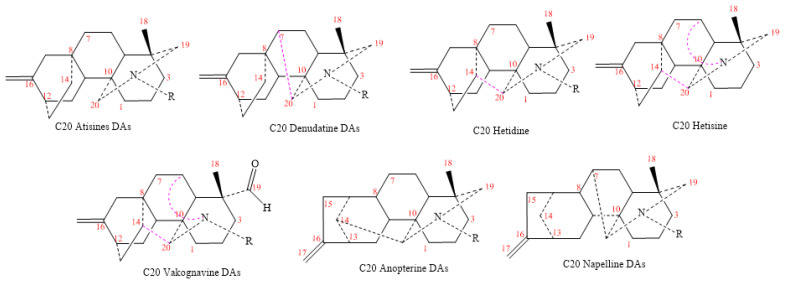
DAs-C_20_ classes.

DAs-C_20_ are more complex compounds than C_18_ and C_19_. They are tetracyclic diterpenes with a 20-carbons skeleton; a *Trans* ring connects between C19 and C20. Most DAs-C_20_ isolated from *Delphinium* and classified as atisine, denudatine, hetisine, hetidine, anopterine, napelline, and vakognavine (Figure 12).

#### 2.3.1. C_20_-Atisines Class

**Figure 13:**
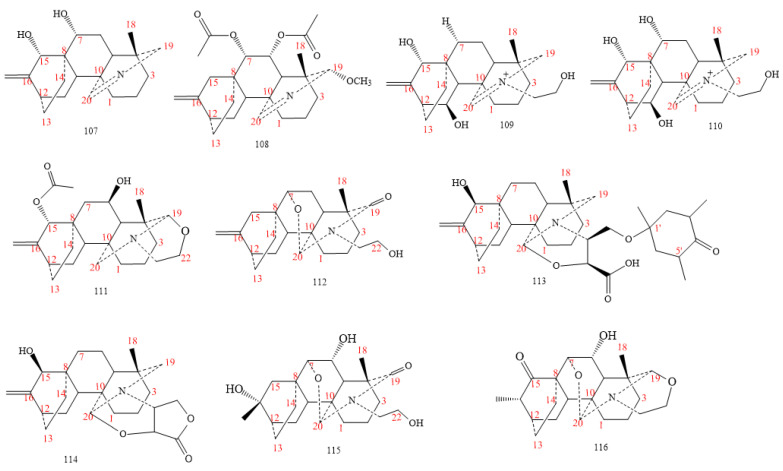
C_20_-atisines DAs.

Atisines are DAs isolated from different species of the genera *Aconitum*, *Delphinium* and *Spiraea* [7,8]. Whereas spirimines A and B (**107** and **108** in Figure 13) result from *Spiraea japonica* var. *acuminata* [77]. DA-**108** shows a methoxy group on C19 [77], whereas leucostomines A and B (**109** and **110** in Figure 13) exhibit a quaternary ammonium hydroxyethyl group. Compounds **111–113** reveal an oxazolidine ring, and compound **112**, a trimethyl-oxocyclohexyloxy group. DAs **114–116** exhibit an O–C–N unit between C7 and C_20_, and structure **116** shows a carbonyl group at C15 [78,79,80].

#### 2.3.2. C_20_-Denudatine Class

**Figure 14:**
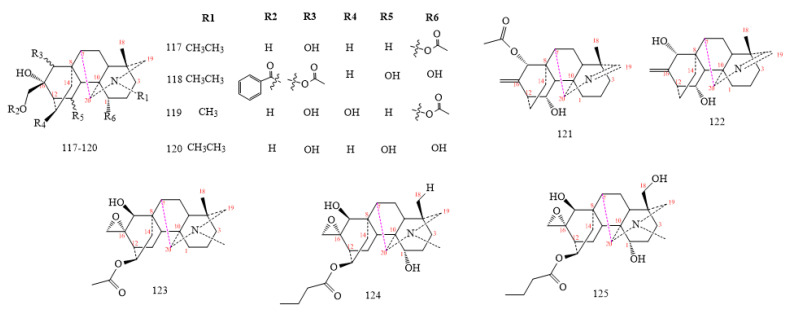
C_20_-denudatine DAs.

Most of the denudatine DAs (compounds **81–89** in Figure 14) originate from *Aconitum spp*, except DAs **123–124** obtained from the whole herb of *Delphinium anthriscifolium* var. *savatieri* [81]. A hydroxyl group and an oxygenated group are respectively on C16 and C17 in DAs **117–120**. Finally, an epoxy group between C16 and C17 is visible in DAs **123–125** [82,83,84,85,86,87,88,89,90,91].

#### 2.3.3. C_20_-Hetisine Class

**Figure 15:**
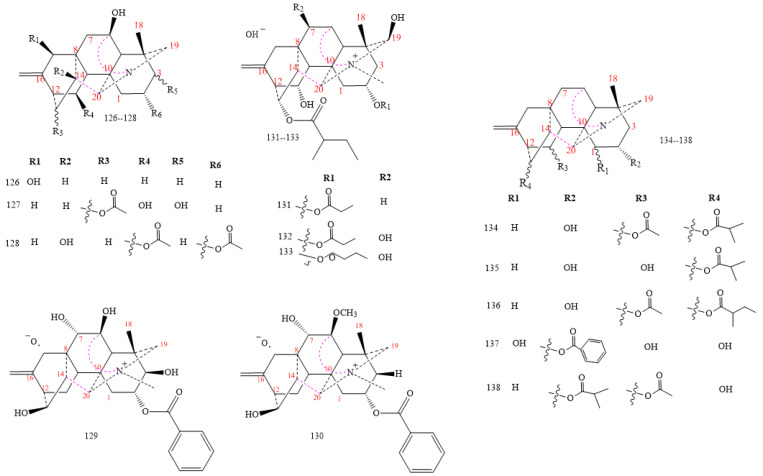
C_20_-hetisine DAs.

Hetisines are the most prominent C_20_-DAs members (structures **126–138** in Figure 15). Most of them isolated from *Aconitum* spp. and *Delphinium* spp. [7,8], and include hydroxyl or methoxide groups in C6 and C3 as shown by structures **126–130** [43]. DAs **126–129** exhibit an OH group on C6, whereas compound **130** brings a methoxide. An a-oriented OH group at C3 is characteristic of most C_20_-DAs; however, compound **129** possesses a b-oriented OH group [92,93,94]. Propionyloxy in C13 and 2-methyl butyryloxy moieties in C2 and a quaternary N base characterise compounds **131–133** obtained from the lateral roots of *A. carmichaelii*. Compounds **134–138** lose an oxygen group in C11 and C13 [67,95,96,97,98,99,100].

#### 2.3.4. C_20_-Hetidine Class

**Figure 16:**
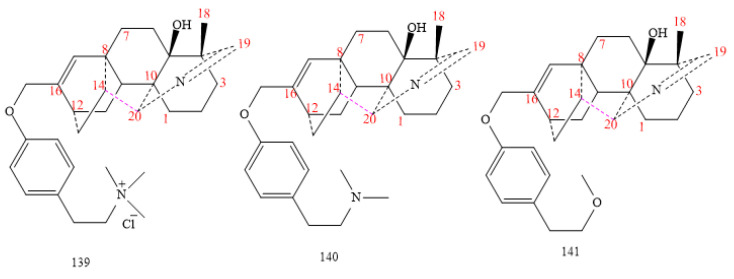
C_20_-hetidine DAs.

The smallest group in the hetidine classification consists of three compounds (**139–141** in Figure 16) [99,101,102]. It is distinguished by the presence of the N=CH group, an endocyclic double bond, and a hydroxyl at C5 in all hetidine-DAs [97,98,99,101]. Rotundifosine F (structure **139** in Figure 16) exhibits a cardicine chloride in C17, whereas the DA **140** shows the hordenine group in the same position, and DA **141** brings a (2-methoxyethyl)-benzene ethanol moiety [99].

#### 2.3.5. C_20_-Vakognavine Class

**Figure 17:**
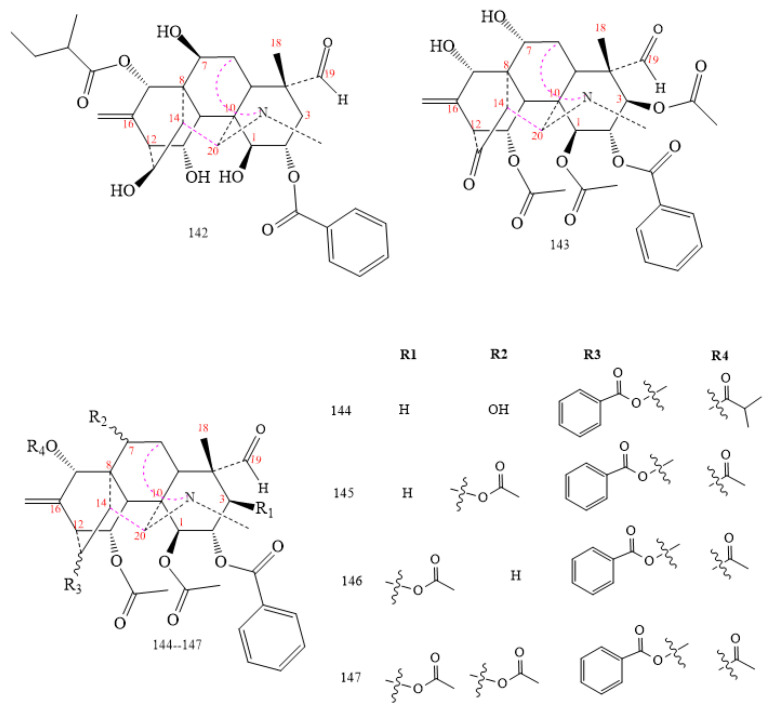
C_20_-vakognavine Das.

Most vakognavine DAs (**142–147** in Figure 17) come from *Aconitum* and *Delphinium*. Structurally, they have a rare double bond between C16 and C17, an aldehyde group in C19, and a characteristic N–Me group [56,93,99,103,104,105].

#### 2.3.6. C_20_-Napelline Class

**Figure 18:**
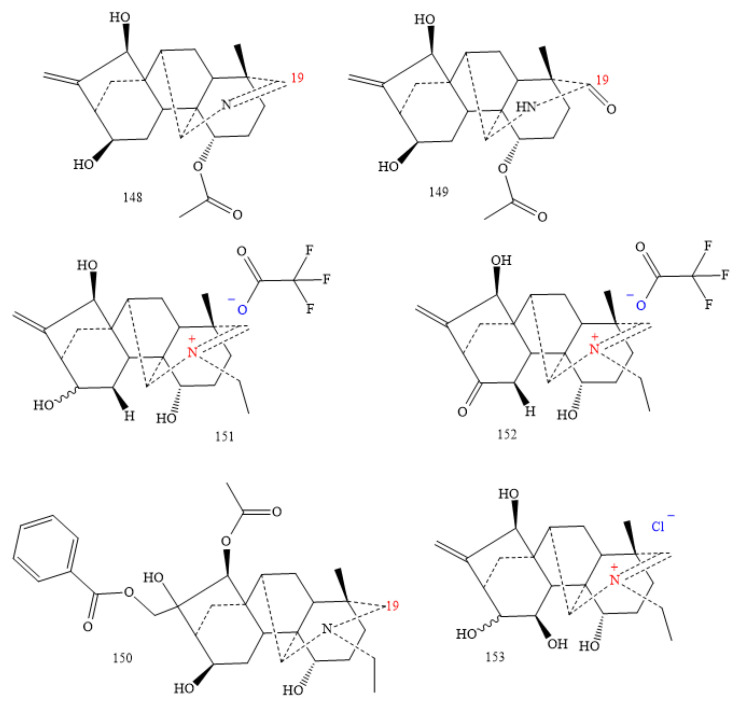
C_20_-napelline DAs.

There are few alkaloids in this class (**148–153** in Figure 18). DA **148** have an N=C19 and an endocyclic double bond; DA **149** a lactam fragment [73,106,107]. Aconicarmichinium A tri-fluoroacetate, aconicarmichinium B trifluoroacetate, and aconicarmichinium C chloride (**151–153**), obtained from the alcohol iminium salts of *A. carmichaelii* [107], complete the class.

#### 2.3.7. C_20_-Anopterine Class

**Figure 19:**
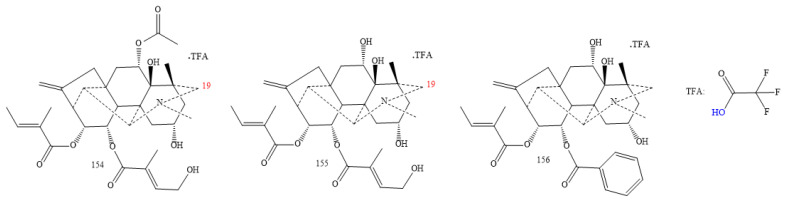
C_20_-anopterine DAs.

DAs in this classification come from *Anopterus/Anopterus macleayanus* species (**154–156** in Figure 19). All anopterine DAs are similar; they have two hydroxyl groups, an N–Me and an endocyclic double bond [108]. They differ only the substituent in C11; compounds **154** and **155** show a hydroxymethyl butenoate, whereas the DA-**156** exhibits an O-benzoyl group [108].

#### 2.3.8. C_20_-Rearranged Classes

**Figure 20:**
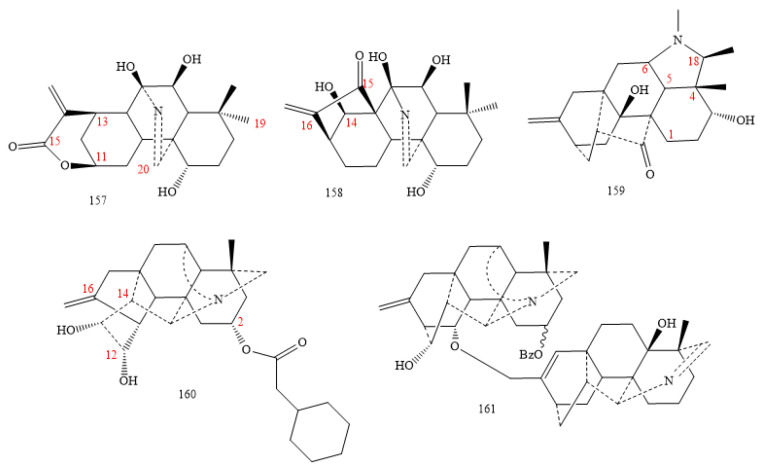
C_20_-rearranged DAs.

They are new C_20_-DAs (**157–160** in Figure 20) with rearranged carbon skeletons.

Kaurine A and B (**157**, **158** in Figure 20) come from *Isodon rubescens*. These two compounds show a 7,20-aza-*ent*-kaurane skeleton instead of a 19,20. Moreover, the DA-**157** exhibit a lactone between C11 and C16 [74,88,109,110].

Compound **159** is isolated from *D. grandiflorum*. Compared to the hetisine class skeleton, the bond between the N atom and C17 was open due to forming a five-member ring, including C4, C5, C6, C18, and the N atom [109].

DA **160** is obtained from the roots of *Delphinium trichophorum*. Its skeleton contains a rearranged C-ring, a pentacyclic structure, and is not hexacyclic, as in a hetisane class [111,112].

Almost all of the C_20_-diterpenoid alkaloids contain oxygenated groups. However, in contrast to the C_19_-diterpenoid alkaloids, C_20_-DAs possess the following distinctive features [113,114,115,116,117,118,119,120,121,122,123,124,125,126,127,128,129,130,131,132,133,134,135,136,137,138,139,140,141,142,143,144,145,146,147,148,149,150,151,152,153,154,155,156,157]:

(i) Most of them do not contain a methoxy group in their structures as C_19_-DAs [108];
(ii) Some alkaloids contain an acetoxy group or benzoyloxy ester group, or both, and do not include other ester groups [56,93];
(iii) Most C_20_-DAs possess exocyclic methylene, and many of them have a secondary hydroxyl function in the allylic position [109,157];
(iv) Few atisine and hetidine-type alkaloids contain N,O- mixed acetal/ketal units [77,78,99,101].

### 2.4. Bis-Diterpenoid Alkaloids

**Figure 21:**
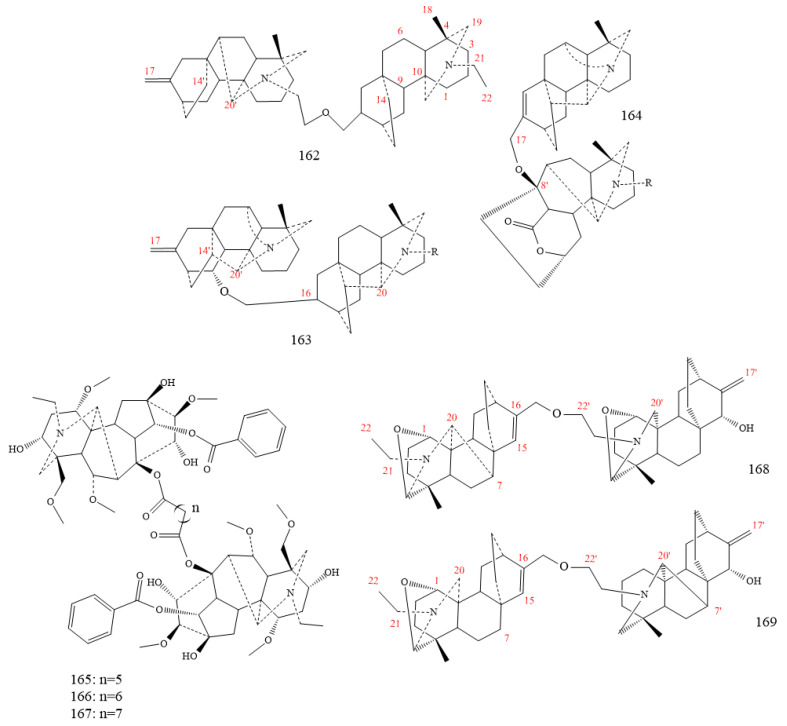
Bis-diterpenoid alkaloids.

Structurally, Bis-DAs (**162–169** in Figure 21) are classified into three classes, atisine–denudatine (**162** in Figure 21), hetidine–hetisine (**163** in Figure 21), and heteratisine–hetidine (**164** in Figure 21). The atisine–denudatine consists of an atisine-type and a denudatine-type C_20_-DA, characterised by an O-ether linkage between atisine and denudatine. Hetidine–hetisine comprises a hetidine-type and a hetisine-type C_20_-DA with an oxygen atom linking hetidine and hetisine in the compound. Heteratisine–hetidine links a lactone-type C_19_-DA and a hetidine-type C_20_-DA [154,155,156,157,158].

## 3. Marine Diterpenoid

Natural products of marine origin have become progressively substantial lead structures for drug discovery [159,160,161,162,163,164,165,166]. However, their structural variety often distinguishes them from products obtained from plants [159]. In this context, the scant availability of material from natural sources often poses a significant limitation to their utilisation.

Diterpenoids obtained from soft corals of the genus *Xenia* show a vast range of biological activities such as antiproliferative [160], antiangiogenic [161], or bactericidal [162] effects.

**Figure 22:**
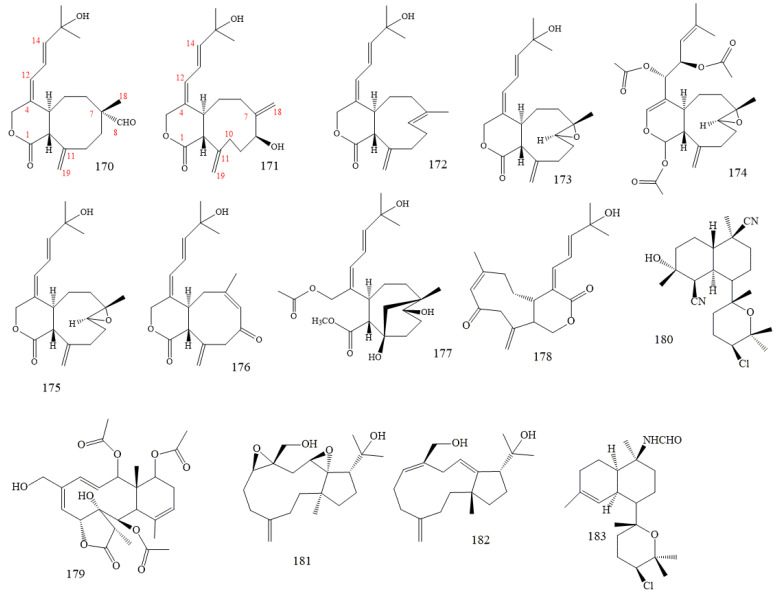
Marine diterpenoids.

The dichloromethane extract from the Formosan soft coral *Xenia blumi* showed significant cytotoxicity to A549 (human lung adenocarcinoma), HT-29 (human colon adenocarcinoma), and P-388 (mouse lymphocytic leukaemia) cell cultures [163,164,165]. Bioassay-guided fractionations of this extract resulted in the isolation of eight new *Xenia*-diterpenoids, blumiolide-A (**170** in Figure 22), blumiolide-B (**171** in Figure 22), 9-deoxy-isoxeniolide-A (**172** in Figure 22), 9-deoxy-7,8-epoxy-isoxeniolide-A (**173** in Figure 22), 9-deacetoxy-7,8-epoxy-13-epi-xenicin (**174** in Figure 22), 9-deoxy-7,8-epoxy-xeniolide-A (**175** in Figure 22), blumiolide-C (**176** in Figure 22), and blumicin-A (**177** in Figure 22) [167,168,169].

*Xenia*-diterpenoid blumiolide C (**178** in Figure 22), isolated from *X. blumi*, exhibits a potent in vitro antiproliferative activity (ED50 values of 1.5 µm and 0.6 µm against the human colon cancer cell line HT-29 and the mouse P-388 leukaemia line, respectively). Structurally, blumiolide C is distinct from most *Xenia*-diterpenoids because of the presence of a *Z*, rather than the commonly found *E*, double bond as part of the nine-membered ring [160,162].

Pachyclavulide B (**179** in Figure 22), isolated from the Okinawan soft coral, *Pachyclavularia violacea*, is a briarane-type diterpenoid containing eight chiral centres and a highly oxygenated tricyclic system [168]. It exhibits moderate growth-inhibitory activity against cancer cells (SNB-75) of the central nervous system [169].

Kalihinol A (**180** in Figure 22), isolated from the Guamanian marine sponge, *Acanthella* sp., is a richly functionalised tricyclic diterpenoid with isocyano and hydroxyl tetrahydropyranyl and chlorine functions [170]. Biological activity, including antimicrobial [170,171,172], antifungal [170,171,172,173,174,175,176], cytotoxic [174], anthelmintic [173,174,175,176,177], and antifouling [178,179,180,181,182], have been reported. Kalihinol A, obtained from the Okinawan sponge, *Acanthella* sp., strongly inhibits proliferation of the malaria parasite, *Plasmodium falciparum* (EC50 1.2 × 10^−9^ M), and express a remarkable selective index (SI 317), defined as the ratio of FM3A cell cytotoxicity to *P. falciparum* [183,184].

Stolonidiol (**181** in Figure 22) and stolonitriene (**182** in Figure 22) are dolabellane-type diterpenoids isolated from the Okinawan marine soft coral, *Clavularia* sp. [185,186]. Most dolabellane-type diterpenoids possess trans-bicyclo tetradecane and exhibit antimicrobial, antitumor, and antiviral activity [187,188]. Stolonidiol is unique for multiple biological activities and expresses potent cytotoxic activity toward P388 leukaemia cells (IC50 0.015 µg mL^−1^) and ichthyologic activity toward killifish, *Oryzias latipes* (minimum lethal concentration: 10 µg mL^−1^) [185].

Kalihinane-type diterpenoid possessing *cis* or *trans*-decalin and tetrahydropyran or tetrahydrofuran as its fundamental skeleton is a highly functionalised marine diterpenoid bearing isocyano, isothiocyanate, formamide, hydroxy, and (or) chlorine groups [189,190,191,192]. Most kalihinane-type diterpenoids exhibit antimicrobial [171,172,189], antifungal [172,175,189], cytotoxic [174], anthelmintic [175,189], antifouling [190], and antimalarial [191] activities.

Kalihinene X (**183** in Figure 22), isolated from the Japanese marine sponge, *Acanthella cavernosa*, is a formamide kalihinane-type diterpene with *cis*-decalin chlorinated tetrahydropyran moieties [190]. Kalihinene X inhibits the attachment and metamorphosis of cyprid larvae of the barnacle, *Balanus amphitrite*, with EC50 of 0.49 µg mL^−1^, which does not show toxicity at this concentration [192].

## 4. Toxicity

Regardless of the broad domain of ‘DAs’ biological activities obtained from *Aconitum* and *Delphinium* plants, DA plants and their compounds are cardiotoxins and potent neurotoxins, despite being evaluated as decorative plants [113,114,115,116,117,118,119].

Toxic DAs mainly affect the central nervous system and the heart, with gastrointestinal side effects. Overdose can lead to death due to the development of ventricular arrhythmias and cardiac arrest [114,119,120]. With the ubiquitous tradition of using DAs as herbal medicines, often disguised as ornamental plants, poisoning cases are notoriously widespread [119,121].

‘DAs’ toxicity is mainly due to the diester diterpene alkaloids (C_19_-Aconitine class), which exhibit two ester groups, an acetyl moiety on C8 and a benzoyl\anisoyl moiety on C14 [114,122]. Therefore, the de-esterification of C_19_-Aconitine DAs reduces their toxicity. For example, the mono-ester diterpene alkaloid benzoylaconine is 200-fold less toxic than aconitine [114]. Furthermore, alkaline hydrolysis of acetyl and benzoyl in aconitine produces aconine (alcohol amine diterpenoid alkaloid), which is less than 1000-fold as toxic as aconitine [122].

In general, the *Aconitum* roots used in traditional medicines follows specialised processing methods, such as soaking, boiling, or hydrolysing; this causes a decrease in aconitine derivatives toxicity (benzylaconine or aconine) [147,150]. When comparing the proportion of aconitine in raw chuanwu to processed chuanwu (soaked or boiled), the balance of aconitine in the raw material is more remarkable. For this reason, the exposure to poisoning is higher when using raw chuanwu [119].

The cardiotoxicity and neurotoxicity of aconitines are in virtue of their actions on the voltage-sensitive sodium channels of the cell membranes of excitable tissues, including the myocardium, nerves, and muscles. Aconitine binds to open sensitive, high-voltage sodium channels, causing continuous sodium channel activation, becoming resistant to excitation. The electrophysiological mechanism of induction of arrhythmias due to delayed post-depolarisation and early post-depolarisation is triggered [114,119,121,122,123].

Aconitine ‘DAs’ arrhythmic properties are part of its cholinergic (anticholinergic) effects mediated by the vagus nerve. Aconitine has a positive inotropic effect by prolonging sodium’s influx during the action potential [114,122].

It has antihypertensive and bradycardia actions by virtue of the activation of the ventral nucleus in the hypothalamus. By acting on voltage-sensitive sodium channels in axons, aconitine inhibits neuromuscular transmission by reducing acetylcholine’s induced quantitative release. On the other hand, aconitine DAs can cause severe contractions of the ileum by releasing acetylcholine from the posterior node cholinergic nerves [114,122].

Studies conducted on the effect of aconitine in mice concluded that it induces cell death by promoting excess Ca^2+^ in the ventricular muscle cells, causing disruption of the Na^+^/Ca^2+^ exchange system and reducing the regulation of the sarco-endoplasmic network of Ca^2+^-ATPase [124,125].

Three diterpene mono-ester alkaloids (MEA) and three diterpene di-ester alkaloids (DEA), tested on fish for cardiac toxicity, revealed how acetate in the C8 position of DEA contributes most to cardiac toxicity [126,127,128].

Unfortunately, there is no specific treatment for *Aconitum* poisoning. In contrast, supportive cardiovascular therapy is usual in poisoning cases [114,122].

## 5. Bio-Activities of DAs

### 5.1. Analgesic Activities

Opioids, salicylates, propionic acid derivatives, oxicam, and other non-steroidal anti-inflammatory drugs, usually used to control pain, have harmful side effects in gastrointestinal damage by inhibiting prostaglandin production in addition to the potential for addiction and adverse effects on the nervous system to opioid users [129].

Over the past ten years, studies have examined the effect of plant parts and alkaloids derived from them, such as *A. carmichaelii* [130], *Aconitum weixiense* [30], *Aconitum bulleyanum* [131], *Aconitum baikalensis* [132], and *Aconitum brachypoumi* [133], which has seen them used as analgesics [20,21,30,72,130,131,132,134,135,136,137].

Investigations on the effectiveness of analgesics obtained from C_18_- and C_19_-DAs showed that aconitine and lappaconitine affect sodium channels. Aconitine inhibits nerve conduction by continuous depolarisation, while lappaconitine may block Na+ channels and act as a local anaesthetic [129,138].

Lappaconitine (C18-DAs) shows pain-relief properties. However, lappaconitine sulfate, obtained by the modification of lappaconitine, exerts a more noticeable analgesic action than lappaconitine, which is poorly soluble in water [139,140].

Studies on the analgesic activity of C_19_-DAs demonstrated that compound **60** in Figure 5b, extracted from *A. carmichaelii*, exerts an analgesic effect on mice when used in acetic acid with a non-toxic dose of 0.5 mg/kg of body weight [141].

Compounds **100** in Figure 10 and **101** in Figure 11, administered in acetic acid using doses of 1.0, 0.3, and 0.1 mg/kg, showed a weak analgesic effect on mice using the higher amount of 1 mg/kg, with a pain suppression rate of 78.34%, whereas the rate was less than 20% for compounds **98** and **99** in Figure 10 [21]. The lack of the methoxy group in C6, as for compounds 94 and 95, seems to exert a fairly noticeable effect on the analgesic activity, whereas the presence of a methoxyl group in C1, as for the compounds 98 and 99, significantly decreases the activity [21].

Other C19-DAs exhibit analgesic effects with low toxicity as guiwuline (compound **90** in Figure 9) [72], bulleyaconitines A, foresaconitines, and yunaconitines [131].

The structure–activity relationship (SAR) analysis revealed the fundamental structures necessary for observing the analgesic activity of the C_19_-DAs. For example, substituents in C8 should be either the acetoxyl or ethoxyl group, a tertiary amine is essential in the cyclohexane ring, and substituents in C14 different from an aromatic ester would reduce the effectiveness. Furthermore, the hydroxylation at C15 is requisite to undergo bioactivation [5,135].

The characteristic skeletons, showing low toxicity in C_20_-DAs, encouraged researchers to conduct studies on their analgesic effects. In contrast to the substantial toxicity of C_18_-DAs and C_19_-DAs, C_20_-DAs may be effective candidate drugs for the management of pain treatments. In addition, the sulfonated compound (**157** in Figure 20), extracted from the lateral roots of *A. carmichaelii*, also showed a significant analgesic activity [142].

### 5.2. Anti-Inflammatory Activities

NSAIDs (salicylates, acetic acid derivatives, profenes, oxycamates, pyrazolidine derivatives, selective cyclooxygenase-2 inhibitors, and phenamic acids) are the most commonly used analgesics and anti-inflammatory drugs. They have many side effects on the digestive and nervous systems [115]. Based on studies conducted on C_19_-DAs extracted from *Aconitum* and *Delphinium*, diterpenoid alkaloids can interact with neurotransmitters, making them good candidates as anti-inflammatory drugs [30,37,55,70,105,129,130,143,144,145,146].

Compound **144** in Figure 17 inhibits the activity of cyclooxygenase-2 (COX-2) with inhibitory concentration (IC_50_) nearly equal to that of acetylsalicylic acid (29.75 µM and 29.30 µM, respectively); this is what makes it a possible alternative to aspirin [105].

The activity of compound **55** in Figure 5b and compound **87** in Figure 8 on inhibiting NO production in lipopolysaccharide cells (LPS) stimulated the macrophage cell line RAW 264.7, with a behaviour similar to dexamethasone. IC_50_ values were 7.46 ± 0.89 µM and 8.09 ± 1.31 µM for compounds **87** and **55**, respectively, and 8.32 ± 1.45 µM for dexamethasone [68,70]. Swatinine (compound **64** in Figure 6, obtained from *Aconitum baikalense* has an anti-inflammatory activity similar to indomethacin, with an inhibition rate of 38.71% and 42.02%, respectively [55]. Therefore, given their particular activity, various DAs can provide good resources for exploring promising anti-inflammatory drugs.

Bulleyanines A and B (**168** and **169** in Figure 21, respectively), two novel compounds, were isolated from *Aconitum bulleyanum*. Compound A showed a marked effect on anti-inflammatory activity with an inhibition rate of 74.60% (40 μmol L^−1^), compound B showed as inactive, as compared to positive control dexamethasone (78.70%) at 100 μg mL^−1^ [158].

### 5.3. Antimicrobial Activities

Several researchers demonstrate the antimicrobial activity of some DAs. For example, sinchiangensine (compound **59** in Figure 5b) has potent antibacterial activity against *Staphylococcus aureus* with minimum inhibitory concentration (MIC) value 0.147 mmol mL^−1^; furthermore, lipodeoxyaconitine (analogue of sinchiangensine) is active against the same bacterium with MIC value 0.144 mmol mL^−1^ [144].

Some C_20_-vakognavine compounds, e.g., carmichaedine (compound **144** in Figure 17), show activity against *Bacillus subtilis* with MIC of 8 mmol mL^−1^ [104]. Besides, some aconitine-type DAs such as vilmorine D, vilmorrianine A, and yunaconitine exhibit antibacterial activity against *S. aureus* and *B. subtilis* [145].

Compound **50A** in Figure 5b, obtained from the roots of *Aconitum duclouxi*, also show antibacterial activity against *B. subtilis* with an MIC of 147.73 mmol L^−1^; moreover, compounds **50A** and **50B** show antifungal activity against *Candida albicans* with MIC of 51.84 and 128 mg mL^−1^, respectively [146,147].

Additionally, aconicaramide, extracted from the lateral roots of *A. carmichaelii*, displays equinoctial antibacterial activity against *Macrococcus caseolyticus*, *Staphylococcus epidermidis*, and *S. aureus* (MIC 200, 400, and 800 mg mL^−1^, respectively) [84].

Oleracein E demonstrated antibacterial activity against *S. aureus*, *M. caseolyticus*, *Klebsiella pneumonia*, and *Streptococcus pneumoniae* (MIC: 50, 200, 200, and 200 mg mL^−1^, respectively) [84].

Extensive laboratory experiments are helpful promoters for the preparation of new antimicrobial formulations.

### 5.4. Antioxidant Activities

Diterpenoid alkaloids showed auspicious 1,1-diphenyl-2-picrylhydrazyl (DPPH)-like scavenging activity. Aconitine-type C_19_-DAs could be suitable antioxidants because of their ability for binding to metal ions [105]. Swatinine compounds (**64** and **73** in Figure 6) offered an effective DPPH radical scavenging ratio of 65.3% and 63.4%, respectively, at 1 µM, whereas butylated hydroxytoluene (standard antioxidant) inhibited to 92.1% at the same concentration [55]. These results indicate that C_19_-DAs could also offer new antioxidant agents, selecting substances with lower toxicity in this group.

### 5.5. Cytotoxic Activity

Various ‘DAs’ anticancer activities have been widely studied from different parts of *Aconitum*, *Consolida*, and *Delphinium* in the last decade [148]. The most effective natural DAs with anticancer properties in *Aconitum* were C_19_-DAs and some derivatives of C_20_-DAs. SAR analysis showed that DA activity increased in correspondence with simple structural modification of these compounds, but their anticancer mechanisms need further studies.

Researchers examined many newly obtained DAs against the lung cancer cell lines, A549. Compounds **59** in Figure 5b [144], **135** [96,97] and **137** [96] in Figure 15, and **160** [96] and **161** [101] in Figure 20 showed appreciable cytotoxicity toward the A549 with IC_50_ < 20 µM.

Other compounds (**1, 3, 4**, and **6** in Figure 1; **22** in Figure 5) were active against liver cancer cell line HepG2 [105], and compound **144** in Figure 17 showed perfect activity against HepG2 with IC_50_ of 3.65 µM [105]. In contrast, DA-**59** (Figure 5b) [144] and compound **161** (Figure 20) showed IC_50_ of 9.18 µM and 18.52 µM against liver cancer cell line SMCC-7721 [101].

DA-**153** (Figure 18) has a strong effect against human prostate carcinoma with IC_50_ of 3.1 µM [108,149]; furthermore, DA-**112** (Figure 13) shows a significant action against human breast adenocarcinoma MCF-7 cell line with IC_50_ 3.16 µM [79].

Compounds **51** [150] and **59** [144] (Figure 5b), and **161** (Figure 20) [101,151], characterize an anticancer activity vs. leukaemia cell lines HL-60, and DAs **51–55** (Figure 5b) exerted potent action against line K562 [150].

SAR of antitumor DAs indicates that the number and position of the hydroxyl and ester groups in C_19_-DAs may play an essential role in cytotoxicity, especially substitutions in C1, C3, C6, and C8 [50,53,92,105,144,148,152,153].

Three new bis-DAs derived from genus *Aconitum* (**165–167** in Figure 21) present remarkable cytotoxic activity in vitro against lung cancer A-549, colon cancer HCT-15, and breast cancer MCF-7 cells; their IC50s were <28 µM [154].

## 6. Conclusions

Over the past decade, more than 300 DAs were discovered and extracted from plants, particularly *Aconitum*, *Delphinium*, and *Consolida* genera.

Structurally, DAs derived from four isoprenyl ‘units’ condensation subdivide into more than 45 classes based on their central structure arrangement and different substituent. These compounds display a broad area of pleasant chemical properties and biological activity, such as analgesic, anti-inflammatory, antimicrobial, cytotoxic activity, and toxic effects. Their toxic effect is manifested in the nervous and cardiovascular systems, acting as potent neurotoxins and cardiotoxins. The toxicity of C_18_-DAs and C_19_-DAs groups has justified their development into new therapeutic drugs, except glycosidic DAs, which have additional sugar moieties in their structures that facilitate their water solubility unlike the other DA groups. This observation gives future hope to discovering new chemical compounds with low toxicity and useful bio-activity in the aqueous extracts of alkaloids with SAR similar to C_19_-DAs. The complex nature of the diterpenoid-alkaloids’ SAR suggests the need for an accurate knowledge of individual compound properties to discover further safe and valuable applications of novel bioactive compounds.

The ‘researchers’ competition, turned to deeper study of C_20_-DAs after a SAR analysis, displayed their chemical structure diversity and their little toxicity compared to C_19_-DAs. In addition, their classification into seven groups with different SARs facilitates the search for biologically active molecules and potential new drugs.

Many research efforts, oriented to studying the anti-inflammatory, analgesic, and anticancer activity of DAs, highlighted that numerous C_19_-DAs and C_20_-DAs have noticeable effectiveness. The C_20_-hetisine class showed the highest possibilities with the lowest toxicity among the other DAs. For this reason, the hetisine compounds may be good starters for developing novel anticancer drugs using alkaloids.

## Data Availability

Not applicable.
